# Author Correction: A comprehensive characterization of the cell-free transcriptome reveals tissue- and subtype-specific biomarkers for cancer detection

**DOI:** 10.1038/s41467-022-30329-0

**Published:** 2022-05-04

**Authors:** Matthew H. Larson, Wenying Pan, Hyunsung John Kim, Ruth E. Mauntz, Sarah M. Stuart, Monica Pimentel, Yiqi Zhou, Per Knudsgaard, Vasiliki Demas, Alexander M. Aravanis, Arash Jamshidi

**Affiliations:** GRAIL, Inc., Menlo Park, CA USA

**Keywords:** Cancer, Cancer screening

Correction to: *Nature Communications* 10.1038/s41467-021-22444-1, published online 21 April 2021.

In the original version of this Article there was an error in the Data Availability Statement. A sentence providing details about the restrictions for granting access to the data generated in this Article ‘Access to the data will be restricted to non-commercial entities’ was omitted. This has been corrected in the pdf and HTML versions of the Article.

