# Supplementary material for: A comprehensive characterization of the cell-free transcriptome reveals tissue- and subtype-specific biomarkers for cancer detection
Source: Nat Commun. 2021 Apr 21;12:2357. doi: 10.1038/s41467-021-22444-1 (PMC8060291; doi:10.1038/s41467-021-22444-1)
Supplement: Supplementary file 1 — Supplementary Information [file 41467_2021_22444_MOESM1_ESM.pdf]

Supplementary Materials

Supplementary Information

Preanalytical testing

We set out to determine the effect of different preanalytical factors on cfRNA in plasma, such as blood collection tube type, shipping temperature of blood tubes, long-term storage of plasma, and freeze-thaw cycles of plasma samples. Lysis of white blood cells (WBC) during sample collection, transport, and storage can confound gene expression estimates generated from RNA-seq, so we created a metric to measure the cfRNA fraction in any given sample and detect WBC lysis. We compared 7 paired WBC/cfRNA samples obtained from Discovery Life Sciences (Huntsville, AL). This sample set contains both cancer and healthy samples, and each sample set was enriched using our targeted RNA-seq assay using a custom panel of 508 cancer-related genes. Collapsed read counts were generated using STAR, and only genes targeted by the panel were considered. DESeq2 was used to identify differentially expressed genes controlling for patient effects. Only genes with a false discovery rate (FDR) <0.01 and with a minimum 4× expression change were considered. A total of 44 genes were found to be overexpressed in WBCs and 32 were found to be overexpressed in cfRNA. In each preanalytical study, the log-transform of the sum of the cfRNA-specific gene count for each sample was compared across conditions to determine the effect of different preanalytical parameters on cfRNA recovery.

Blood collection tube type

We set out to compare preservation of cfRNA in blood collected in Streck cell-free DNA tubes (catalog # 218996) vs Streck cell-free RNA tubes (catalog # 218975). We collected 4 tubes of whole blood from each of 7 donors. Blood was drawn into two Streck RNA tubes and two Streck DNA tubes for each donor. Whole blood was processed to plasma, and the plasma pooled according to tube type (Streck RNA vs Streck DNA) across patients, creating two pooled plasma fractions. After pooling, the samples were aliquoted into 16 x 8 mL fractions (8 tubes Streck RNA plasma and 8 tubes Streck DNA plasma) and extracted in parallel using the QIAamp Circulating Nucleic Acid kit.

We prepared RNA-seq libraries for each aliquot of extracted cfRNA using the protocol described in the main text. RNA-seq libraries were enriched using a custom panel of 508 cancer-related genes, and each library was sequenced to saturation (~200M reads per sample). A comparison of collapsed gene counts for a given tube type provided a measure of assay reproducibility, while a comparison of collapsed gene counts across the different tube types provided a measure of tube-to-tube differences. Samples collected in the same tube type were more closely correlated with each other than with samples drawn in a different tube type (Supplementary Fig. 1), suggesting that technical replicates were highly reproducible, but that blood collection tube type affected the representation of transcripts in the cfRNA fraction.

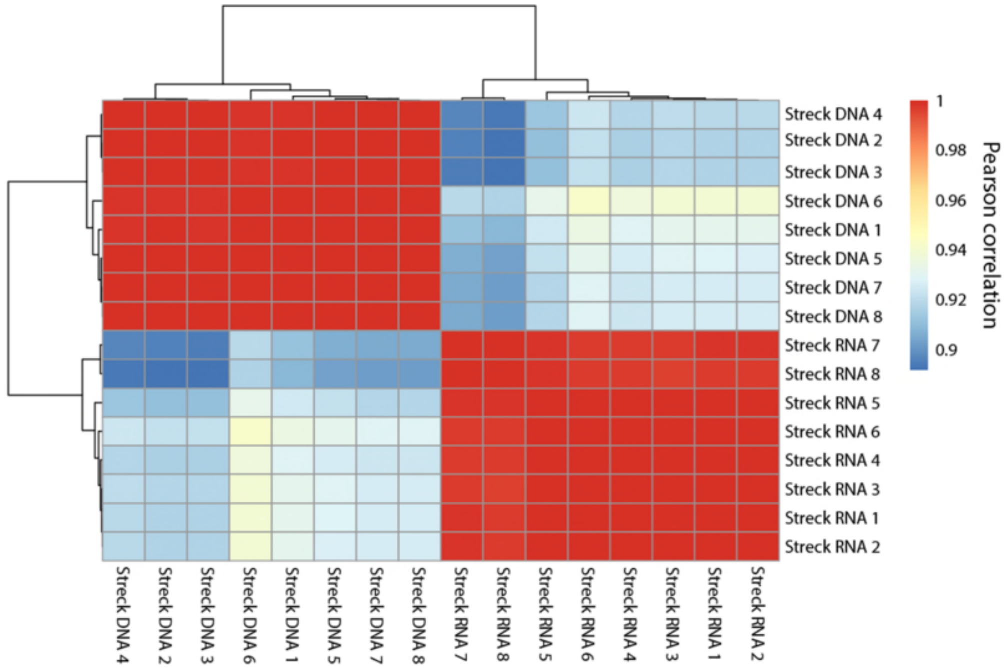

Supplementary Fig. 1. Pairwise correlations as a function of blood collection tube type. Pairwise Pearson correlation of collapsed gene counts for samples collected in different blood collection tube types.

A differential expression analysis using DESeq2 indicated that 862 genes (of 63,664 total genes from Gencode v19 comprehensive gene annotation) were differentially expressed according to blood collection tube type ($P < 0.01$, as determined by DESeq2) (Supplementary Fig. 2a). In general, we found that blood-related transcripts, such as hemoglobin genes (*HBA1*, *HBA2*, *HBB*) were more highly expressed in samples collected in Streck RNA tubes compared to the samples collected in Streck DNA tubes, suggesting increased hemolysis in Streck RNA tubes (see highlighted genes in Supplementary Fig. 2b). The apparent increase of mitochondrial genes in Streck DNA tubes (Supplementary Fig. 2b) further supports this hypothesis, as red blood cells lack mitochondria, leading to a relative reduction in the normalized expression of mitochondrial genes in Streck RNA upon increased hemolysis.

a

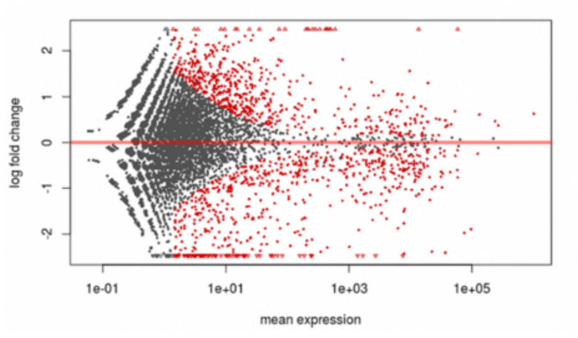

b

Higher in Streck DNA tubes

Gene	Base Mean	Log Fold Change
MT-ND4	481.9851	2.722392
MT-RNR2	58633.3445	2.758584
MT-ND5	441.8311	2.663842
MT-ND1	504.4979	2.943488
MPL	9608.395	1.563541
MT-ND2	432.134	2.821219
HLA-B	791.7665	1.813875
MT-CYB	284.6496	2.68949
MT-RNR1	13554.3624	2.615123
MT-CO3	256.3742	2.807092

Higher in Streck RNA tubes

Gene	Base Mean	Log Fold Change
HBA1	1382.3384	-2.866163
CDH1	1686.6945	-3.141931
HBA2	2711.1871	-2.733049
TFRC	22308.6291	-2.375259
TERC	7921.1984	-2.426289
PIM1	36849.0478	-2.405195
HBB	7531.5347	-2.735123
RNY4	240.906	-4.065336
ICOSLG	445.3151	-2.29739
BCL2L1	74572.1088	-1.974307

Supplementary Fig. 2. Effect of blood collection tube type on cfRNA gene expression. a Log fold change in expression as a function of blood collection tube. Differentially expressed genes (P < 0.01, as determined by DESeq2) are highlighted in red. **b** Top 10 differentially expressed genes by tube type. Hemoglobin genes are highlighted in red.

Motivated by these differences in gene expression, we analyzed the effect of blood collection tube type on both cfRNA-enriched genes and WBC-enriched genes and determined that samples collected in the Streck DNA tube had increased coverage for cfRNA genes (Supplementary Fig. 3a), and that samples collected in the Streck RNA tube had increased coverage for blood cell genes (Supplementary Fig. 3b). These results both support the conclusions of the individual gene expression analysis (i.e. that samples drawn into Streck DNA tubes show less evidence of hemolysis compared to samples collected in Streck RNA tubes) and validate our approach to measuring the effect of different preanalytical parameters through the analysis of specific gene sets. They also support our selection of the Streck DNA tubes for the preservation and analysis of cfRNA.

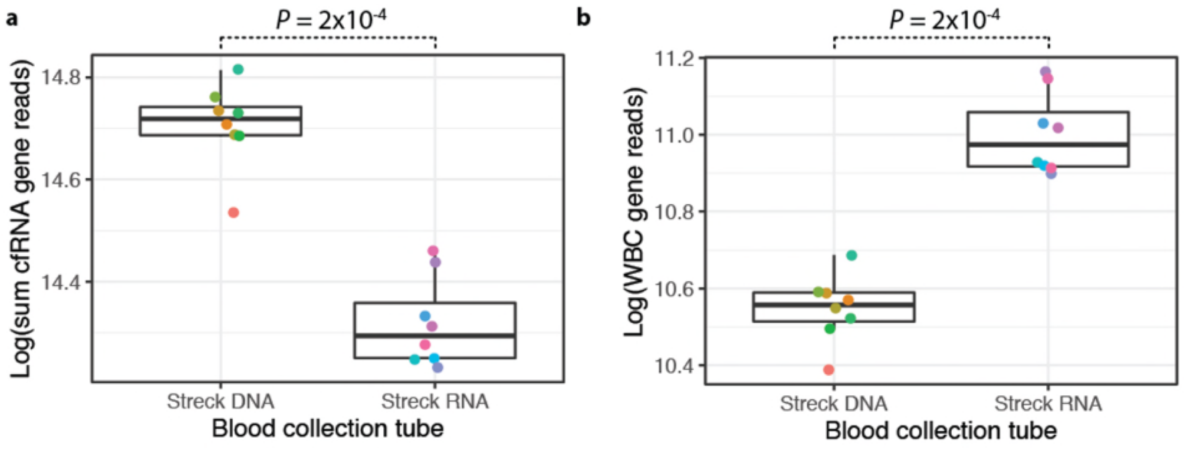

Supplementary Fig. 3. Effect of blood collection tube type on cell-free RNA recovery from plasma. The log transform of either **a** cfRNA-specific gene count or **b** WBC-specific gene count plotted as a function of blood collection tube type. 8 replicates (colored dots) are represented for each tube type. *P* values are calculated from the two-sided Wilcoxon rank-sum method. Boxplots indicate the 25% (lower hinge), 50% (horizontal line), and 75% quantiles (upper hinge), with whiskers that indicate observations outside the hinge ± 1.5 x interquartile range (IQR). Outliers (beyond 1.5 x IQR) are plotted individually.

Shipping temperature of blood tubes

Blood from 12 non-cancer donors (8 × 10 mL tube from each donor) was ordered from StemExpress and collected in Streck Cell Free DNA blood collection tubes and transported to GRAIL (Menlo Park, CA) via courier at ambient temperature within 4 hours of draw. On arrival at GRAIL, the tubes were held at 22°C until 24 ± 4 hours had elapsed since the time of draw. The tubes were then randomized for draw order, divided into 4 groups, and held at the different temperatures (6°C, 14°C, 22°C, and 37°C) for 8 hours to simulate temperature excursions during transit. After 8 hours, all the tubes were returned to holding at 22°C until 48 hours had elapsed

since the time of draw, simulating the maximum shipping time for the majority of samples in the CCGA study. Plasma isolation began 48 ± 4 hours after blood draw for all samples, at which point 2 aliquots of plasma from each donor were pooled and cell-free nucleic acid (cfNA) was extracted the same day using our standard protocol. The extracted cfNA was frozen and stored at –80°C. The following day, cfNA was DNase treated and quantified using the High Sensitivity RNA Fragment Analyzer (Agilent), converted into DNA libraries, and enriched using our custom panel.

The log-transform of cfRNA-specific yield at each temperature was compared to the baseline temperature (22°C), with multiple hypothesis adjustment using Dunnett's test. A significant difference was observed in cfRNA-specific gene count between 37°C and 22°C: there was ~28% decrease in yield at 37°C (95% confidence interval [CI], 4.5-48%), suggesting a loss of cfRNA material at high temperatures. There was no significant change in cfRNA yield at lower temperatures (6°C and 14°C) compared to 22°C.

Based on this result, we repeated the study with 12 non-cancer donors (8 × 10 mL tube from each donor), but changed the temperature excursions to 30°C, 33°C, and 35°C for 4 hours to better simulate real-world high-temperature excursions during shipping. This study showed that there was no statistically significant difference in cfRNA coverage up to 33°C, but that coverage increased slightly at 35°C (34% increase [95% CI, 0.7-78%]). The increase in coverage at 35°C, compared to the decrease in coverage at 37°C in the first shipping temperature study, is likely due to batch effects for cfRNA extraction and processing, which were performed on a later date by a different set of clinical operators.

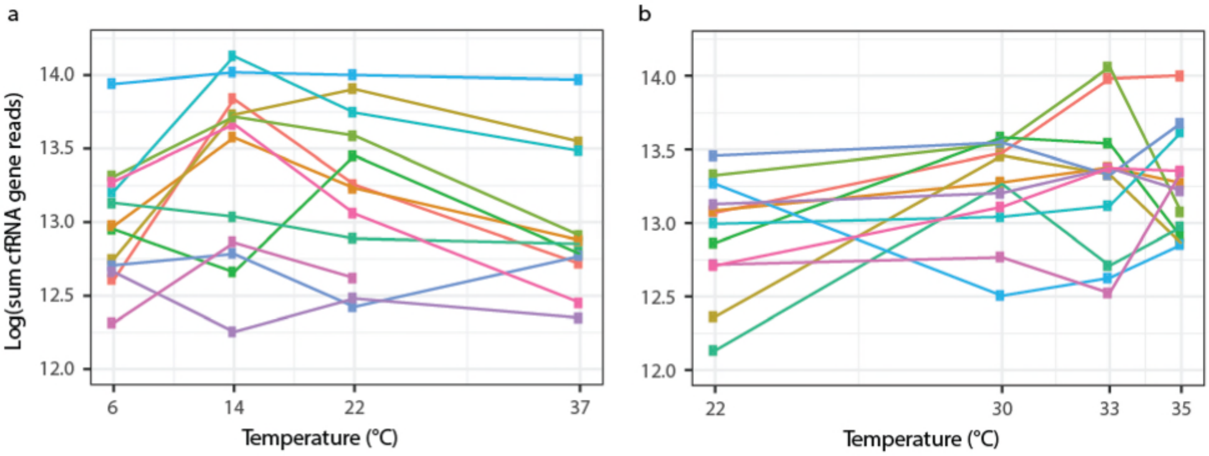

Temperatures	Mean	Lower CI	Upper CI	*P* value
37 – 22°C	-27.6%	-44.9%	-4.8%	0.016
6 – 22°C	-20.5%	-39.0%	3.8%	0.110
14 – 22°C	11.4%	-14.7%	45.3%	0.663

Temperatures	Mean	Lower	Upper	*P* value
30 – 22°C	26.2%	-5.1%	67.8%	0.138
33 – 22°C	32.6%	-0.3%	76.4%	0.053
35 – 22°C	33.9%	0.7%	78.2%	0.043

Supplementary Fig. 4. Effect of shipping temperature on cell-free RNA recovery from plasma. The log-transform of the cfRNA-specific gene count for each patient (n=12) was compared across temperatures ranging between **a** 6 to 37°C and **b** 22 to 35°C. Each colored line represents a single non-cancer donor. Mean and 95% confidence interval limits (lower and upper CI) for percent change in the log-transform of cfRNA-specific gene count between the test conditions and the reference condition (22°C) are shown in the tables below the graphs. *P* values were determined using Dunnett's test. cfRNA, cell-free RNA.

Long-term plasma storage

Plasma from 84 non-cancer CCGA donors were used to evaluate the effects of extended plasma storage on cfRNA. Samples from 3 sets of subjects (groups 1, 2, and 3) collected approximately 0, 3, and 6 months (± 28 days) from the start of the experiment were used for the study. Each set consisted of 28 subjects, and each subject had 6 tubes available to facilitate testing over 3 time

points with 2 tubes per time point. Samples from each set were processed at 0 months, 3 months, and 6 months. This approach allowed for assessment of cfRNA stability up to 12 months.

At each time point, 2 tubes of plasma for each of 84 subjects were removed from storage at –80°C, thawed, extracted, and DNase treated using our standard protocol. Each cfRNA sample was run on the Fragment Analyzer to assess the extraction yield and fragment length integrity of the cfRNA, converted into DNA libraries, and enriched through a custom targeted panel. We modeled the effect of plasma storage on the log-transformed cfRNA-enriched count using a mixed-effects regression model where storage time was designed as a fixed effect and the patient is designated as a random effect. Storage time did not have a significant effect on cfRNA-specific gene counts over 12 months in storage (Supplementary Fig. 5).

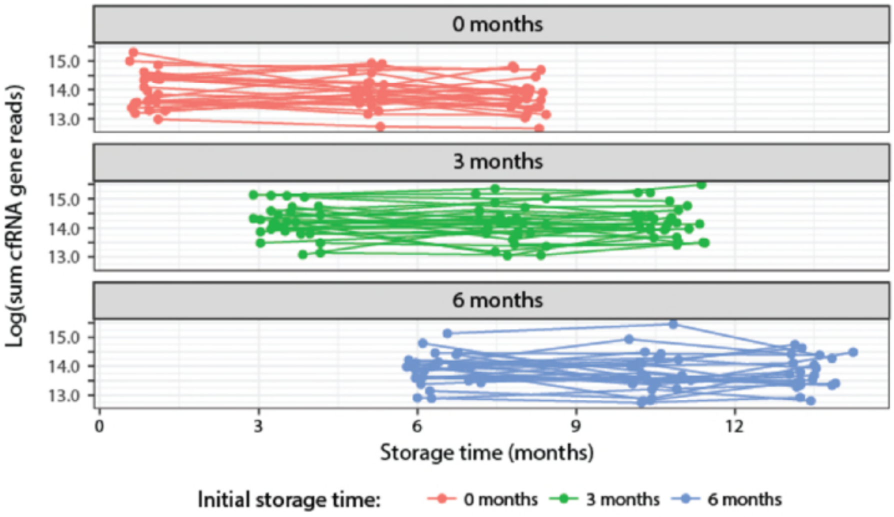

Storage group	Mean	Lower CI	Upper CI	P value
Grp1_3mo – Grp1_0mo	-4.9%	-26.5%	23.0%	0.996
Grp1_6mo – Grp1_0mo	-18.6%	-37.3%	5.7%	0.191
Grp2_3mo – Grp1_0mo	25.0%	-16.5%	87.1%	0.550
Grp2_6mo – Grp1_0mo	19.4%	-20.2%	78.6%	0.767
Grp2_9mo – Grp1_0mo	29.6%	-13.4%	94.0%	0.388
Grp3_6mo – Grp1_0mo	-9.4%	-39.5%	35.6%	0.983
Grp3_9mo – Grp1_0mo	-21.2%	-47.7%	18.9%	0.502
Grp3_12mo – Grp1_0mo	-16.6%	-45.1%	26.9%	0.779

Supplementary Fig. 5. Effect of plasma storage on cell-free RNA recovery. Sample groups 1, 2, and 3 each consist of a distinct set of 28 subjects from whom blood was collected at different times (0, 3, and 6 months, respectively) at the start of the study. Group 1 donors are sampled at 0, 3, and 6 months from the date of collection. Group 2 donors are sampled at 3, 6, and 9 months from the date of collection. Group 3 donors are sampled at 6, 9, and 12 months from the date of collection. Mean and 95% confidence interval limits (lower and upper CI) for percent change in the log-transform of cfRNA-specific gene count between storage subgroups and the reference group (Grp1_0mos) are shown in the table below the graph. *P* values were determined using Dunnett's test. cfRNA, cell-free RNA.

Freeze-thaw cycles

Plasma samples from 14 healthy donors were collected to evaluate the effects of freeze-thaw cycles on cfRNA for performance in a targeted enrichment assay. Twelve tubes of blood were collected from each donor in Streck cfDNA blood collection tubes. On arrival at GRAIL, plasma was pooled within donor and re-aliquoted into 6 replicates per donor. We tested 3 conditions: fresh (no freeze-thaw cycle), 1 freeze-thaw cycle, and 2 freeze-thaw cycles. Each of the 14 donors had 2 experimental replicates per condition. Two replicates were immediately extracted and DNase treated for the fresh condition. The remaining 4 replicates went through freeze-thaw cycles during which they were frozen at –80°C for 24 hours then thawed at room temperature before extraction. Each cfRNA sample was run on the Fragment Analyzer to assess the extraction yield and fragment length integrity of the cfRNA, converted into DNA libraries, and enriched through a targeted panel.

A Dunnett's comparison, where freshly extracted plasma serves as the control, shows that a single freeze-thaw cycle does not result in a statistically significant drop in cfRNA-specific gene coverage (P=0.467). However, there is a statistically significant drop in cfRNA-enriched count when comparing 2 freeze-thaw cycles with freshly extracted plasma ($P<10^{-10}$). The percent change in cfRNA-enriched count for 2 freeze-thaw cycles is –33.3% (95% CI, –39.5% to –26.4%]).

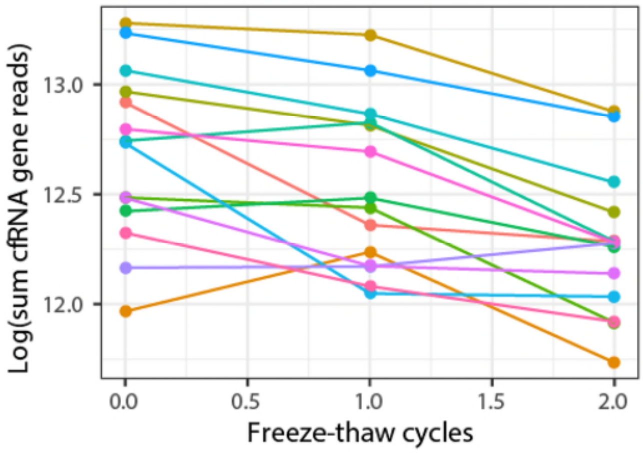

Freeze-thaw group	Mean	Lower CI	Upper CI	*P* value
FT1 – Fresh	-4.6%	-14.0%	5.7%	0.498
FT2 – Fresh	-33.3%	-41.7%	-23.7%	<0.001

Supplementary Fig. 6. Effect of freeze-thaw cycles on cell-free RNA recovery from plasma. The log-transform of the cfRNA-specific gene count for each patient (n=14) was compared across freeze-thaw cycles. Each colored line represents a single non-cancer donor. Mean and 95% confidence interval limits (lower and upper CI) for percent change in the log-transform of cfRNA-specific gene count between samples with 1 or 2 freeze-thaw cycles (FT1 or FT2) and samples without freeze-thaw ("Fresh") are shown in the table below the graph. *P* values were determined using Dunnett's test. cfRNA, cell-free RNA.

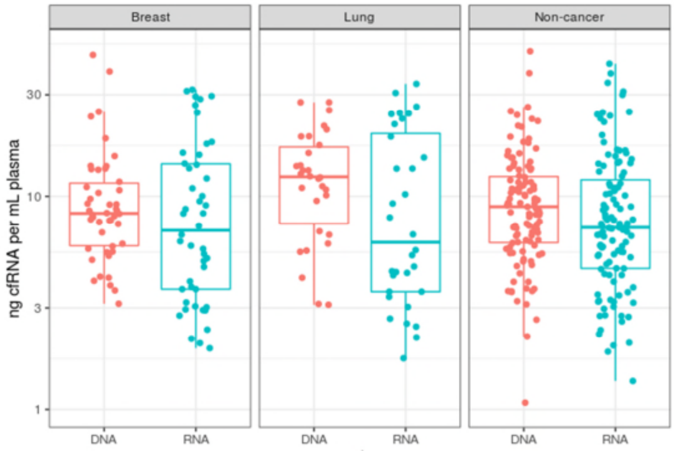

Supplementary Fig. 7. Cell-free DNA and RNA yields by cancer type. Extraction yields from matched plasma sets from patients with cancer (n=47 breast, left panel; n=32 lung, center panel) or without cancer (n=112, right panel). Boxplots indicate the 25% (lower hinge), 50% (horizontal line), and 75% quantiles (upper hinge), with whiskers that indicate observations outside the hinge ± 1.5 x interquartile range (IQR). Outliers (beyond 1.5 x IQR) are plotted individually.

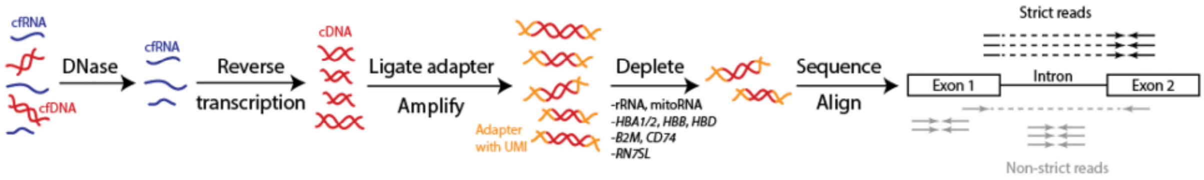

Supplementary Fig. 8. Whole-transcriptome library generation and quantification. The workflow for cfRNA library preparation and gene expression quantification through the use of “strict reads.” cDNA, complementary DNA; cfDNA, cell-free DNA; cfRNA, cell-free RNA; DNase, deoxyribonuclease; HB, hemoglobin; mitoRNA, mitochondrial RNA; UMI, unique molecular identifier.

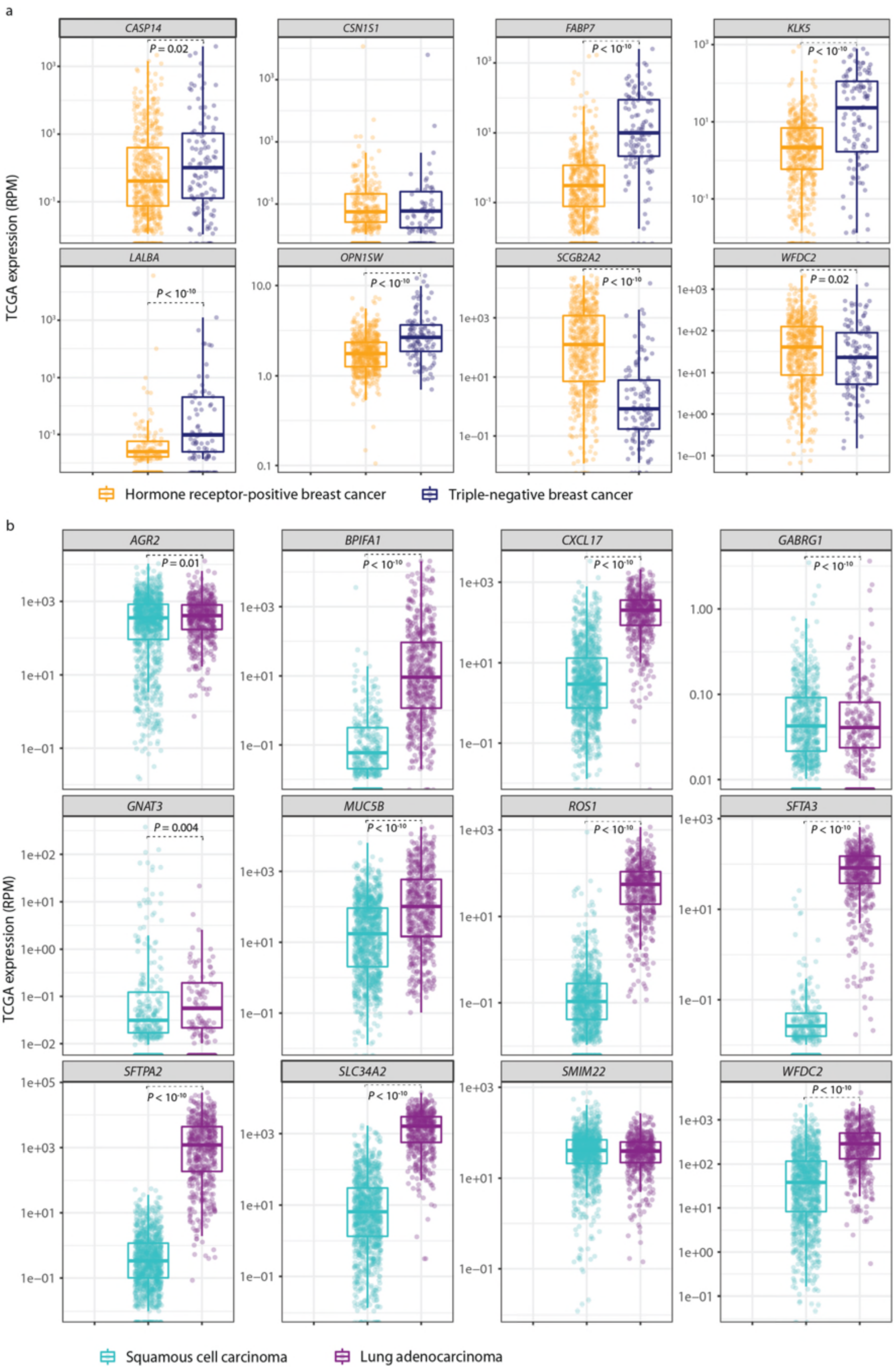
a
CASP14
P = 0.02
CSN1S1
FABP7
P < 10^-10
KLK5
P < 10^-10
LALBA
P < 10^-10
OPN1SW
P < 10^-10
SCGB2A2
P < 10^-10
WFDC2
P = 0.02
TCGA expression (RPM)
Hormone receptor-positive breast cancer
Triple-negative breast cancer
b
AGR2
P = 0.01
BPIFA1
P < 10^-10
CXCL17
P < 10^-10
GABRG1
P < 10^-10
GNAT3
P = 0.004
MUC5B
P < 10^-10
ROS1
P < 10^-10
SFTA3
P < 10^-10
SFTPA2
P < 10^-10
SLC34A2
P < 10^-10
SMIM22
WFDC2
P < 10^-10
TCGA expression (RPM)
Squamous cell carcinoma
Lung adenocarcinoma

Supplementary Fig. 9. Subtype specificity of dark channel biomarker (DCB) genes. Expression of tissue-specific DCB genes in TCGA tissue samples across **a** breast cancer subtypes (n=575 HR+, n=115 triple-negative) and **b** lung cancer subtypes (n=1102 squamous cell carcinoma, n=533 adenocarcinoma). *P* values from the two-sided Wilcoxon rank-sum method indicate significance levels for differential expression between cancer subtypes. Boxplots indicate the 25% (lower hinge), 50% (horizontal line), and 75% quantiles (upper hinge), with whiskers that indicate observations outside the hinge ± 1.5 x interquartile range (IQR). Outliers (beyond 1.5 x IQR) are plotted individually. RPM, reads per million; TCGA, The Cancer Genome Atlas.

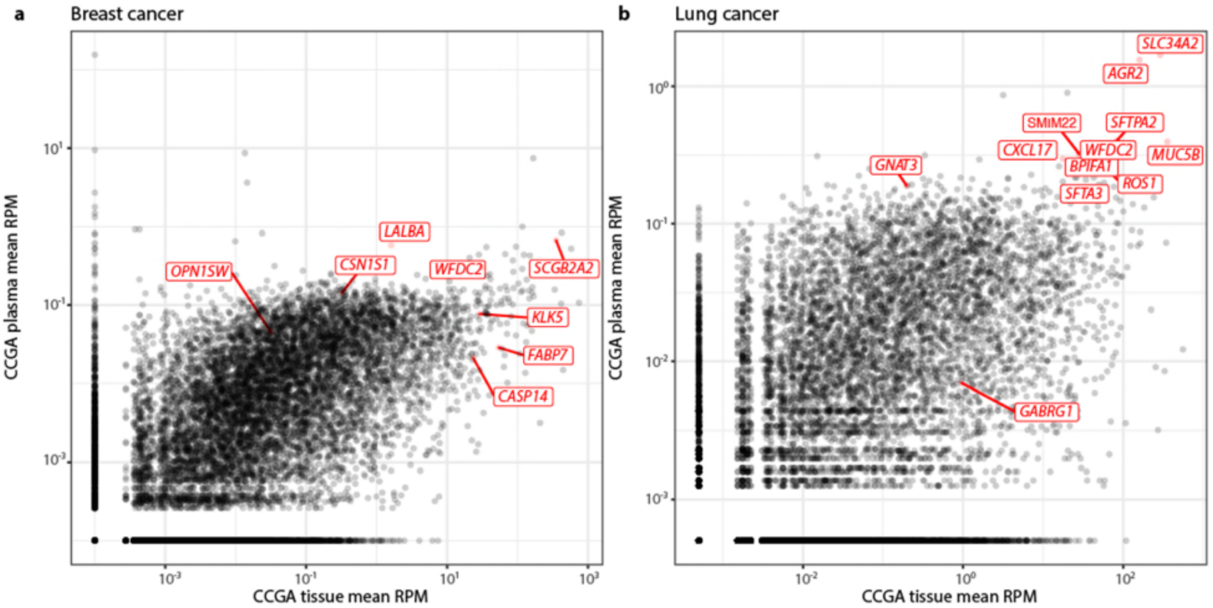

c

	Spearman's ρ breast cancers	Spearman's ρ lung cancers
CCGA tissue vs. CCGA plasma	0.742	0.678
TCGA tissue vs. CCGA tissue	0.631	0.578
TCGA tissue vs. CCGA plasma	0.568	0.509

Supplementary Fig. 10. Dark channel biomarker gene expression levels in plasma are correlated with tumor tissue expression. Correlation of cfRNA gene expression with RNA expression in matched tumor tissue for patients with **a** breast and **b** lung cancer. **c** Spearman's correlation of gene expression between different sample types. CCGA, Circulating Cell-free Genome Atlas study (NCT02889978); cfRNA, cell-free RNA; RPM, reads per million; TCGA, The Cancer Genome Atlas.

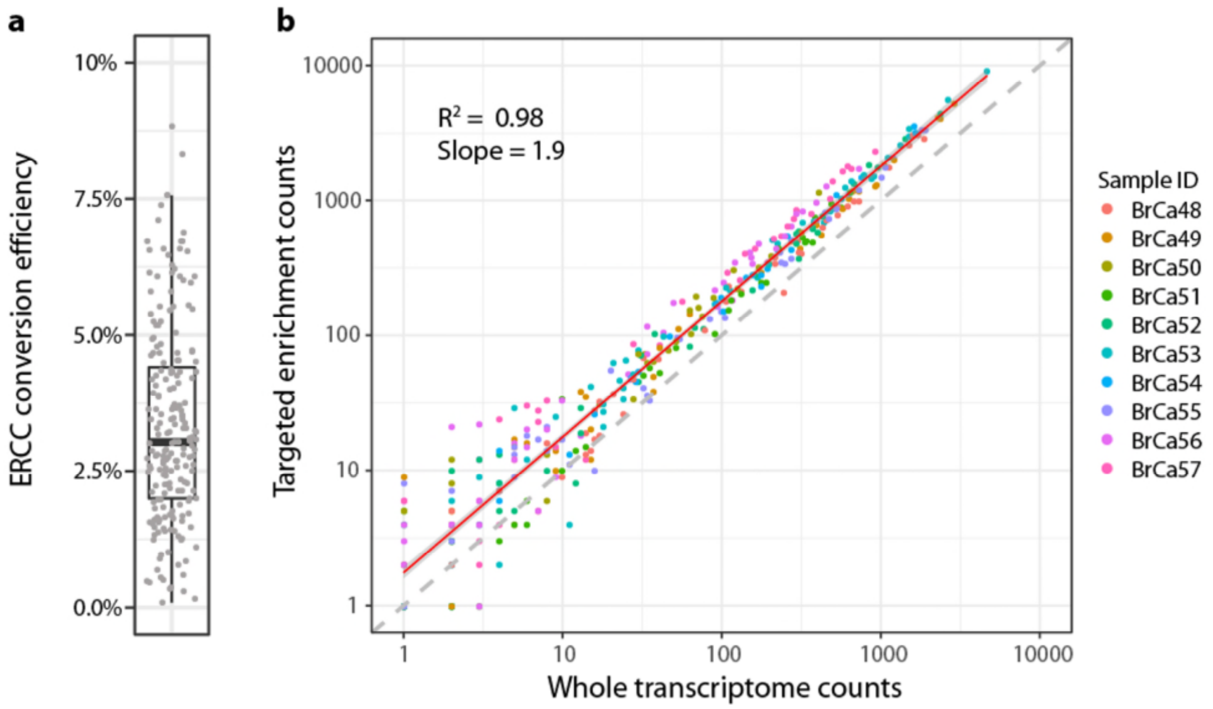

Supplementary Fig. 11. Conversion efficiency for different sequencing library preparation strategies. a The conversion efficiency of each sample (n=165, gray circles) in the whole-transcriptome discovery cohort was monitored using ERCC spike-ins. The mean (SD) conversion efficiency for the whole-transcriptome assay was 4 ± 3%. Boxplot indicates the 25% (lower hinge), 50% (horizontal line), and 75% quantiles (upper hinge), with whiskers that indicate observations outside the hinge ± 1.5 x interquartile range (IQR). Outliers (beyond 1.5 x IQR) are plotted individually. **b** Comparison of strict counts for the targeted enrichment and whole-transcriptome assays for genes within the targeted panel, using a subset of 10 individuals from the breast cancer (BrCa) group of the validation cohort. A linear regression model (red line) showed a 2-fold increase in counts detected with the targeted enrichment assay. The identity line is shown as a dashed grey line. ERCC, External RNA Controls Consortium; SD, standard deviation.

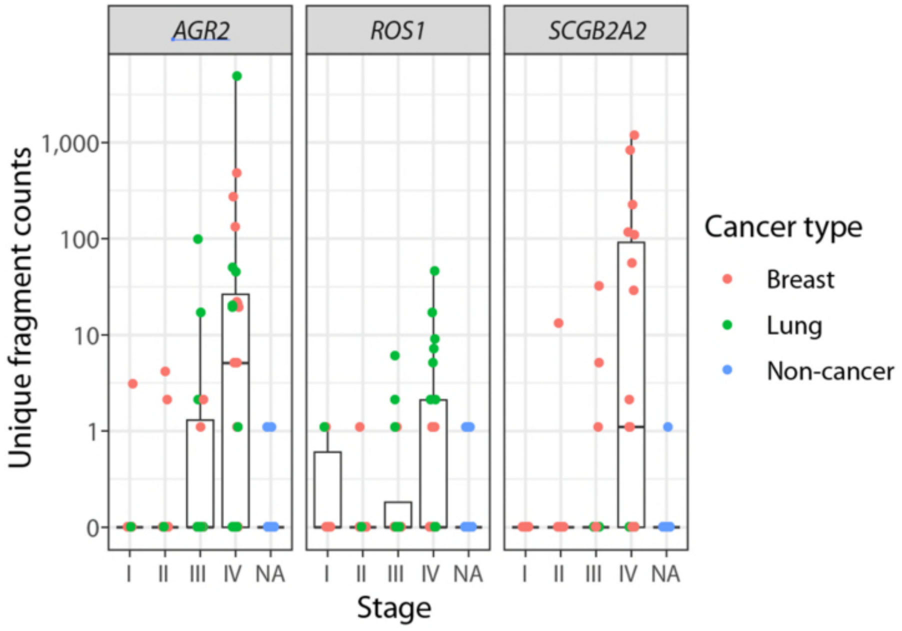

Supplementary Fig. 12. Dark channel biomarker (DCB) expression levels in plasma increase with cancer stage. The number of unique fragment counts for 3 dark channel biomarker (DCB) genes in patients from the validation cohort organized by cancer stage. Solid circles represent individual patients and colors denote cancer type. Data represents 37 breast cancer patients (stages I [5], II [8], III [9], IV [15]), 18 lung cancer patients (stages I [1], II [1], III [7], IV [9]), and 32 non-cancer patients. *AGR2* is present in both breast and lung cancer plasma samples, *ROS1* is specific to lung cancer, and *SCGB2A2* is specific to breast cancer. NA, not available. Boxplots indicate the 25% (lower hinge), 50% (horizontal line), and 75% quantiles (upper hinge), with whiskers that indicate observations outside the hinge ± 1.5 x interquartile range (IQR). Outliers (beyond 1.5 x IQR) are plotted individually.

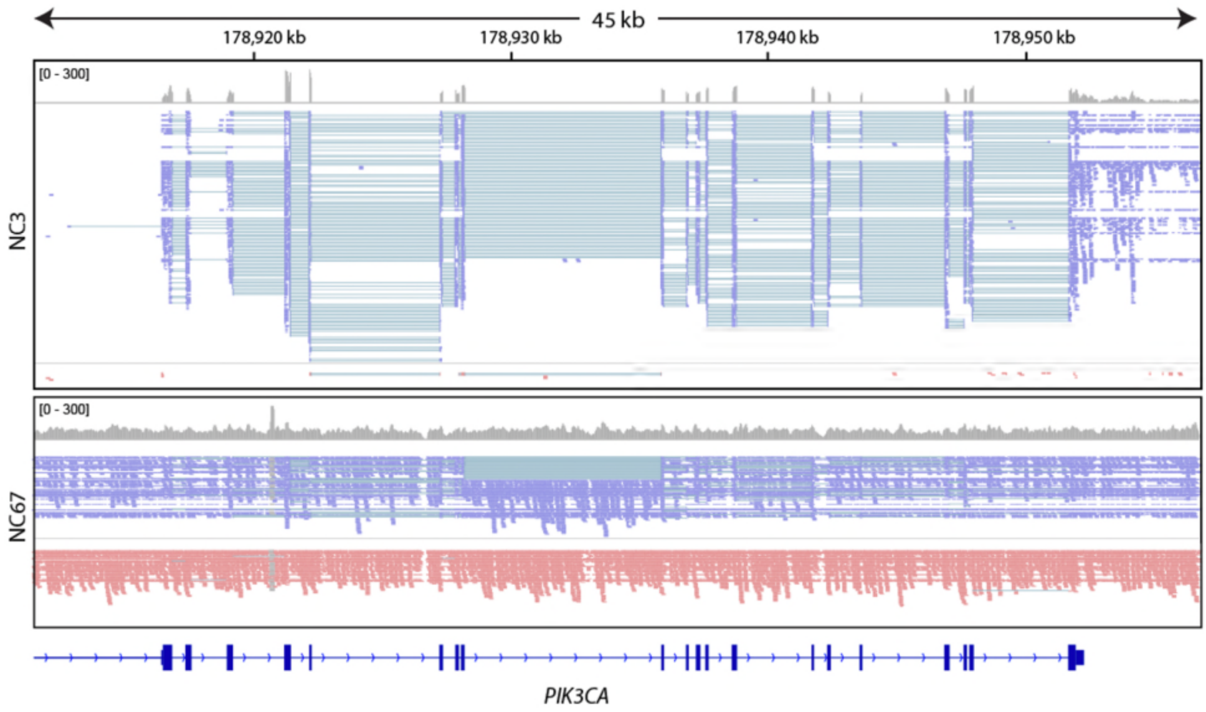

Supplementary Fig. 13. Evidence of DNA contamination in RNA-seq libraries. Reads mapping across a 45kb region of *PIK3CA*, a high-abundance cell-free RNA gene, in a low (NC3, top panel) and high (NC67, bottom panel) DNA contamination sample. Sense (blue) and anti-sense (red) reads are shown. Reads mapping across an intron are connected by a cyan line.

Supplementary Tables

Supplementary Table 1. Sample summary for circulating cell-free genome atlas study discovery cohort

Sample Type	cfRNA (Pass QC)	Tissue (Pass QC)
Breast cancer	47 (46)	40 (40)
Lung cancer	32 (30)	12 (12)
Non-cancer	93 (89)	0
Total	172 (165)	52 (52)

cfRNA, cell-free RNA; QC, quality control.

Supplementary Table 2. Patient demographics for circulating cell-free genome atlas study discovery cohort

	Breast cancer	Lung cancer	Non-cancer
N	47	32	93
Subtype	25 HR+ (14 HR+/HER2-, 11 HR+/HER2+), 5 HR-/HER2+, 14 TNBC, 3 other/missing	11 adenocarcinoma, 10 squamous cell carcinoma, 9 small cell lung cancer, 1 carcinoid, 1 other/missing	
Age, y (median/IQR)	53 (41-60)	66 (60-73)	62 (55-69)
% Female	100%	56%	73%
% Current smoker	2%	34%	8%
% Former smoker	34%	63%	38%
% Non-smoker	64%	3%	55%

HR, hormone receptor; HER2, human epidermal growth factor receptor-2; IQR, interquartile range; TNBC, triple-negative breast cancer.

Supplementary Table 3. Sample summary for circulating cell-free genome atlas study validation cohort

**Stage**	**Breast Cancer (Pass QC)**	**Lung Cancer (Pass QC)**	**Age-Matched Non-Cancer (Pass QC)**
I	5 (5)	1 (1)	NA
II	8 (7)	1 (1)	NA
III	10 (8)	7 (7)	NA
IV	15 (15)	9 (9)	NA
Total	38 (35)	18 (18)	32 (31)

NA, not available; QC, quality control.

Supplementary Table 4. Patient demographics for circulating cell-free genome atlas study validation cohort

	**Breast Cancer**	**Lung Cancer**	**Age-Matched Non-Cancer**
N	38	18	32
Age, y (Median/IQR)	58 (40.5-75.5)	63.5 (54-73)	58 (40.25-75.75)
% Female	100%	67%	87.5%
% Current smoker	8%	22%	3%
% Former smoker	21%	61%	22%
% Non-smoker	71%	17%	72%
% Smoking status unknown	—	—	3%

IQR, interquartile range.

Supplementary Table 5. List of study participants

Sample ID	Smoking Status	Subtype	Matched tissue	QC	Cohort
BrCa1	Former smoker	HR-/HER2+	Not Available	Passed	Discovery (CCGA)
BrCa2	Non-smoker	TNBC	Available	Passed	Discovery (CCGA)
BrCa3	Non-smoker	HR+/HER2+	Available	Passed	Discovery (CCGA)
BrCa4	Non-smoker	TNBC	Not Available	Passed	Discovery (CCGA)
BrCa5	Former smoker	HR+/HER2+	Available	Passed	Discovery (CCGA)
BrCa6	Non-smoker	HR+/HER2-	Available	Failed	Discovery (CCGA)
BrCa7	Non-smoker	Other/missing	Available	Passed	Discovery (CCGA)
BrCa8	Non-smoker	HR+/HER2-	Available	Passed	Discovery (CCGA)
BrCa9	Non-smoker	HR+/HER2-	Available	Passed	Discovery (CCGA)
BrCa10	Non-smoker	HR-/HER2+	Available	Passed	Discovery (CCGA)
BrCa11	Former smoker	Other/missing	Available	Passed	Discovery (CCGA)
BrCa12	Non-smoker	TNBC	Available	Passed	Discovery (CCGA)
BrCa13	Non-smoker	TNBC	Available	Passed	Discovery (CCGA)
BrCa14	Former smoker	TNBC	Not Available	Passed	Discovery (CCGA)
BrCa15	Former smoker	TNBC	Available	Passed	Discovery (CCGA)
BrCa16	Non-smoker	HR-/HER2+	Available	Passed	Discovery (CCGA)
BrCa17	Non-smoker	HR+/HER2+	Available	Passed	Discovery (CCGA)
BrCa18	Non-smoker	HR+/HER2-	Available	Passed	Discovery (CCGA)
BrCa19	Non-smoker	HR+/HER2-	Available	Passed	Discovery (CCGA)
BrCa20	Non-smoker	TNBC	Not Available	Passed	Discovery (CCGA)

BrCa21	Non-smoker	HR+/HER2-	Available	Passed	Discovery (CCGA)
BrCa22	Current smoker	HR+/HER2+	Available	Passed	Discovery (CCGA)
BrCa23	Non-smoker	HR+/HER2-	Available	Passed	Discovery (CCGA)
BrCa24	Former smoker	HR+/HER2+	Available	Passed	Discovery (CCGA)
BrCa25	Non-smoker	HR-/HER2+	Available	Passed	Discovery (CCGA)
BrCa26	Non-smoker	TNBC	Available	Passed	Discovery (CCGA)
BrCa27	Non-smoker	HR+/HER2-	Available	Passed	Discovery (CCGA)
BrCa28	Non-smoker	TNBC	Available	Passed	Discovery (CCGA)
BrCa29	Former smoker	HR+/HER2-	Available	Passed	Discovery (CCGA)
BrCa30	Non-smoker	TNBC	Not Available	Passed	Discovery (CCGA)
BrCa31	Non-smoker	HR+/HER2+	Available	Passed	Discovery (CCGA)
BrCa32	Non-smoker	HR+/HER2+	Available	Passed	Discovery (CCGA)
BrCa33	Former smoker	Other/missing	Available	Passed	Discovery (CCGA)
BrCa34	Non-smoker	HR+/HER2+	Available	Passed	Discovery (CCGA)
BrCa35	Former smoker	HR+/HER2+	Not Available	Passed	Discovery (CCGA)
BrCa36	Former smoker	HR+/HER2+	Available	Passed	Discovery (CCGA)
BrCa37	Former smoker	HR+/HER2-	Available	Passed	Discovery (CCGA)
BrCa38	Former smoker	HR+/HER2-	Available	Passed	Discovery (CCGA)
BrCa39	Former smoker	TNBC	Available	Passed	Discovery (CCGA)
BrCa40	Former smoker	HR+/HER2-	Available	Passed	Discovery (CCGA)
BrCa41	Former smoker	HR+/HER2+	Not Available	Passed	Discovery (CCGA)
BrCa42	Non-smoker	HR-/HER2+	Available	Passed	Discovery (CCGA)

BrCa43	Former smoker	TNBC	Available	Passed	Discovery (CCGA)
BrCa44	Non-smoker	HR+/HER2-	Available	Passed	Discovery (CCGA)
BrCa45	Non-smoker	TNBC	Available	Passed	Discovery (CCGA)
BrCa46	Non-smoker	TNBC	Available	Passed	Discovery (CCGA)
BrCa47	Non-smoker	HR+/HER2-	Available	Passed	Discovery (CCGA)
BrCa48	Non-smoker	Other/missing	Not Available	Passed	Validation
BrCa49	Former smoker	Other/missing	Not Available	Passed	Validation
BrCa50	Non-smoker	Other/missing	Not Available	Passed	Validation
BrCa51	Non-smoker	Other/missing	Not Available	Passed	Validation
BrCa52	Non-smoker	Other/missing	Not Available	Passed	Validation
BrCa53	Non-smoker	Other/missing	Not Available	Passed	Validation
BrCa54	Non-smoker	Other/missing	Not Available	Passed	Validation
BrCa55	Former smoker	Other/missing	Not Available	Passed	Validation
BrCa56	Current smoker	Other/missing	Not Available	Passed	Validation
BrCa57	Non-smoker	HR+/HER2+	Not Available	Passed	Validation
BrCa58	Non-smoker	Other/missing	Not Available	Passed	Validation
BrCa59	Former smoker	Other/missing	Not Available	Passed	Validation
BrCa60	Former smoker	HR+/HER2-	Not Available	Passed	Validation
BrCa61	Non-smoker	TNBC	Not Available	Passed	Validation
BrCa62	Non-smoker	TNBC	Not Available	Passed	Validation
BrCa63	Former smoker	HR+/HER2-	Not Available	Passed	Validation
BrCa64	Non-smoker	HR+/HER2-	Not Available	Passed	Validation

BrCa65	Non-smoker	Other/missing	Not Available	Passed	Validation
BrCa66	Non-smoker	HR+	Not Available	Passed	Validation
BrCa67	Non-smoker	HR+/HER2-	Not Available	Passed	Validation
BrCa68	Current smoker	HR-/HER2+	Not Available	Passed	Validation
BrCa69	Non-smoker	HR+/HER2-	Not Available	Passed	Validation
BrCa70	Non-smoker	HR-/HER2+	Not Available	Passed	Validation
BrCa71	Former smoker	TNBC	Not Available	Passed	Validation
BrCa72	Current smoker	HR+/HER2-	Not Available	Passed	Validation
BrCa73	Former smoker	HR+/HER2-	Not Available	Passed	Validation
BrCa74	Non-smoker	HR+/HER2+	Not Available	Passed	Validation
BrCa75	Non-smoker	HR+/HER2-	Not Available	Passed	Validation
BrCa76	Non-smoker	HR+/HER2+	Not Available	Passed	Validation
BrCa77	Non-smoker	HR+/HER2-	Not Available	Passed	Validation
BrCa78	Non-smoker	Other/missing	Not Available	Passed	Validation
BrCa79	Non-smoker	HR+	Not Available	Passed	Validation
BrCa80	Non-smoker	HR+	Not Available	Passed	Validation
BrCa81	Former smoker	HR+/HER2-	Not Available	Passed	Validation
BrCa82	Non-smoker	HR+/HER2+	Not Available	Failed	Validation
BrCa83	Non-smoker	HR-	Not Available	Passed	Validation
BrCa84	Non-smoker	Other/missing	Not Available	Passed	Validation
BrCa85	Non-smoker	Other/missing	Not Available	Passed	Validation
LuCa1	Current smoker	Small cell lung cancer	Not Available	Passed	Discovery (CCGA)

LuCa2	Former smoker	Adenocarcinoma	Not Available	Failed	Discovery (CCGA)
LuCa3	Former smoker	Adenocarcinoma	Available	Passed	Discovery (CCGA)
LuCa4	Former smoker	Squamous cell carcinoma	Not Available	Passed	Discovery (CCGA)
LuCa5	Former smoker	Small cell lung cancer	Available	Passed	Discovery (CCGA)
LuCa6	Former smoker	Carcinoid	Available	Passed	Discovery (CCGA)
LuCa7	Former smoker	Squamous cell carcinoma	Available	Passed	Discovery (CCGA)
LuCa8	Former smoker	Small cell lung cancer	Not Available	Passed	Discovery (CCGA)
LuCa9	Current smoker	Other/missing	Not Available	Passed	Discovery (CCGA)
LuCa10	Current smoker	Adenocarcinoma	Not Available	Passed	Discovery (CCGA)
LuCa11	Current smoker	Small cell lung cancer	Not Available	Passed	Discovery (CCGA)
LuCa12	Former smoker	Small cell lung cancer	Available	Passed	Discovery (CCGA)
LuCa13	Former smoker	Squamous cell carcinoma	Not Available	Passed	Discovery (CCGA)
LuCa14	Current smoker	Small cell lung cancer	Not Available	Passed	Discovery (CCGA)
LuCa15	Former smoker	Small cell lung cancer	Not Available	Passed	Discovery (CCGA)
LuCa16	Current smoker	Squamous cell carcinoma	Available	Passed	Discovery (CCGA)
LuCa17	Former smoker	Adenocarcinoma	Not Available	Passed	Discovery (CCGA)
LuCa18	Current smoker	Squamous cell carcinoma	Not Available	Passed	Discovery (CCGA)
LuCa19	Former smoker	Squamous cell carcinoma	Available	Passed	Discovery (CCGA)
LuCa20	Current smoker	Small cell lung cancer	Not Available	Failed	Discovery (CCGA)
LuCa21	Former smoker	Squamous cell carcinoma	Not Available	Passed	Discovery (CCGA)
LuCa22	Former smoker	Adenocarcinoma	Not Available	Passed	Discovery (CCGA)
LuCa23	Former smoker	Adenocarcinoma	Not Available	Passed	Discovery (CCGA)

LuCa24	Former smoker	Squamous cell carcinoma	Not Available	Passed	Discovery (CCGA)
LuCa25	Former smoker	Adenocarcinoma	Available	Available	Discovery (CCGA)
LuCa26	Non-smoker	Adenocarcinoma	Not Available	Passed	Discovery (CCGA)
LuCa27	Current smoker	Small cell lung cancer	Available	Passed	Discovery (CCGA)
LuCa28	Former smoker	Squamous cell carcinoma	Not Available	Passed	Discovery (CCGA)
LuCa29	Current smoker	Adenocarcinoma	Not Available	Passed	Discovery (CCGA)
LuCa30	Former smoker	Adenocarcinoma	Not Available	Passed	Discovery (CCGA)
LuCa31	Current smoker	Adenocarcinoma	Available	Passed	Discovery (CCGA)
LuCa32	Former smoker	Squamous cell carcinoma	Available	Passed	Discovery (CCGA)
LuCa33	Former smoker	Adenocarcinoma	Not Available	Passed	Validation
LuCa34	Former smoker	Adenocarcinoma	Not Available	Passed	Validation
LuCa35	Former smoker	Adenocarcinoma	Not Available	Passed	Validation
LuCa36	Non-smoker	Adenocarcinoma	Not Available	Passed	Validation
LuCa37	Current smoker	Adenocarcinoma	Not Available	Passed	Validation
LuCa38	Former smoker	Adenocarcinoma	Not Available	Passed	Validation
LuCa39	Former smoker	Adenocarcinoma	Not Available	Passed	Validation
LuCa40	Former smoker	Adenocarcinoma	Not Available	Passed	Validation
LuCa41	Current smoker	Adenocarcinoma	Not Available	Passed	Validation
LuCa42	Former smoker	Adenocarcinoma	Not Available	Passed	Validation
LuCa43	Current smoker	Adenocarcinoma	Not Available	Passed	Validation
LuCa44	Former smoker	Adenocarcinoma	Not Available	Passed	Validation
LuCa45	Non-smoker	Adenocarcinoma	Not Available	Passed	Validation

LuCa46	Non-smoker	Adenocarcinoma	Not Available	Passed	Validation
LuCa47	Former smoker	Adenocarcinoma	Not Available	Passed	Validation
LuCa48	Current smoker	Adenocarcinoma	Not Available	Passed	Validation
LuCa49	Former smoker	Adenocarcinoma	Not Available	Passed	Validation
LuCa50	Former smoker	Adenocarcinoma	Not Available	Passed	Validation
NC1	Non-smoker	NA	Not Available	Passed	Discovery (CCGA)
NC2	Current smoker	NA	Not Available	Failed	Discovery (CCGA)
NC3	Non-smoker	NA	Not Available	Passed	Discovery (CCGA)
NC4	Non-smoker	NA	Not Available	Passed	Discovery (CCGA)
NC5	Former smoker	NA	Not Available	Passed	Discovery (CCGA)
NC6	Non-smoker	NA	Not Available	Passed	Discovery (CCGA)
NC7	Non-smoker	NA	Not Available	Passed	Discovery (CCGA)
NC8	Non-smoker	NA	Not Available	Passed	Discovery (CCGA)
NC9	Former smoker	NA	Not Available	Passed	Discovery (CCGA)
NC10	Non-smoker	NA	Not Available	Passed	Discovery (CCGA)
NC11	Non-smoker	NA	Not Available	Passed	Discovery (CCGA)
NC12	Non-smoker	NA	Not Available	Passed	Discovery (CCGA)
NC13	Former smoker	NA	Not Available	Passed	Discovery (CCGA)
NC14	Former smoker	NA	Not Available	Passed	Discovery (CCGA)
NC15	Non-smoker	NA	Not Available	Passed	Discovery (CCGA)
NC16	Former smoker	NA	Not Available	Passed	Discovery (CCGA)
NC17	Former smoker	NA	Not Available	Passed	Discovery (CCGA)

NC18	Former smoker	NA	Not Available	Passed	Discovery (CCGA)
NC19	Non-smoker	NA	Not Available	Passed	Discovery (CCGA)
NC20	Non-smoker	NA	Not Available	Passed	Discovery (CCGA)
NC21	Non-smoker	NA	Not Available	Passed	Discovery (CCGA)
NC22	Former smoker	NA	Not Available	Passed	Discovery (CCGA)
NC23	Former smoker	NA	Not Available	Passed	Discovery (CCGA)
NC24	Non-smoker	NA	Not Available	Passed	Discovery (CCGA)
NC25	Former smoker	NA	Not Available	Passed	Discovery (CCGA)
NC26	Former smoker	NA	Not Available	Passed	Discovery (CCGA)
NC27	Non-smoker	NA	Not Available	Passed	Discovery (CCGA)
NC28	Non-smoker	NA	Not Available	Passed	Discovery (CCGA)
NC29	Non-smoker	NA	Not Available	Passed	Discovery (CCGA)
NC30	Former smoker	NA	Not Available	Failed	Discovery (CCGA)
NC31	Former smoker	NA	Not Available	Passed	Discovery (CCGA)
NC32	Former smoker	NA	Not Available	Passed	Discovery (CCGA)
NC33	Non-smoker	NA	Not Available	Passed	Discovery (CCGA)
NC34	Non-smoker	NA	Not Available	Passed	Discovery (CCGA)
NC35	Former smoker	NA	Not Available	Passed	Discovery (CCGA)
NC36	Non-smoker	NA	Not Available	Passed	Discovery (CCGA)
NC37	Former smoker	NA	Not Available	Passed	Discovery (CCGA)
NC38	Non-smoker	NA	Not Available	Passed	Discovery (CCGA)
NC39	Non-smoker	NA	Not Available	Passed	Discovery (CCGA)

NC40	Non-smoker	NA	Not Available	Passed	Discovery (CCGA)
NC41	Former smoker	NA	Not Available	Passed	Discovery (CCGA)
NC42	Non-smoker	NA	Not Available	Passed	Discovery (CCGA)
NC43	Non-smoker	NA	Not Available	Passed	Discovery (CCGA)
NC44	Non-smoker	NA	Not Available	Passed	Discovery (CCGA)
NC45	Non-smoker	NA	Not Available	Passed	Discovery (CCGA)
NC46	Former smoker	NA	Not Available	Passed	Discovery (CCGA)
NC47	Former smoker	NA	Not Available	Passed	Discovery (CCGA)
NC48	Non-smoker	NA	Not Available	Passed	Discovery (CCGA)
NC49	Former smoker	NA	Not Available	Passed	Discovery (CCGA)
NC50	Former smoker	NA	Not Available	Passed	Discovery (CCGA)
NC51	Former smoker	NA	Not Available	Passed	Discovery (CCGA)
NC52	Non-smoker	NA	Not Available	Passed	Discovery (CCGA)
NC53	Non-smoker	NA	Not Available	Passed	Discovery (CCGA)
NC54	Non-smoker	NA	Not Available	Passed	Discovery (CCGA)
NC55	Non-smoker	NA	Not Available	Passed	Discovery (CCGA)
NC56	Non-smoker	NA	Not Available	Passed	Discovery (CCGA)
NC57	Non-smoker	NA	Not Available	Passed	Discovery (CCGA)
NC58	Current smoker	NA	Not Available	Passed	Discovery (CCGA)
NC59	Non-smoker	NA	Not Available	Passed	Discovery (CCGA)
NC60	Non-smoker	NA	Not Available	Passed	Discovery (CCGA)
NC61	Non-smoker	NA	Not Available	Passed	Discovery (CCGA)

NC62	Current smoker	NA	Not Available	Passed	Discovery (CCGA)
NC63	Non-smoker	NA	Not Available	Passed	Discovery (CCGA)
NC64	Former smoker	NA	Not Available	Passed	Discovery (CCGA)
NC65	Current smoker	NA	Not Available	Passed	Discovery (CCGA)
NC66	Former smoker	NA	Not Available	Passed	Discovery (CCGA)
NC67	Non-smoker	NA	Not Available	Failed	Discovery (CCGA)
NC68	Former smoker	NA	Not Available	Passed	Discovery (CCGA)
NC69	Non-smoker	NA	Not Available	Passed	Discovery (CCGA)
NC70	Former smoker	NA	Not Available	Passed	Discovery (CCGA)
NC71	Current smoker	NA	Not Available	Failed	Discovery (CCGA)
NC72	Former smoker	NA	Not Available	Passed	Discovery (CCGA)
NC73	Former smoker	NA	Not Available	Passed	Discovery (CCGA)
NC74	Non-smoker	NA	Not Available	Passed	Discovery (CCGA)
NC75	Non-smoker	NA	Not Available	Passed	Discovery (CCGA)
NC76	Non-smoker	NA	Not Available	Passed	Discovery (CCGA)
NC77	Non-smoker	NA	Not Available	Passed	Discovery (CCGA)
NC78	Non-smoker	NA	Not Available	Passed	Discovery (CCGA)
NC79	Non-smoker	NA	Not Available	Passed	Discovery (CCGA)
NC80	Current smoker	NA	Not Available	Passed	Discovery (CCGA)
NC81	Former smoker	NA	Not Available	Passed	Discovery (CCGA)
NC82	Current smoker	NA	Not Available	Passed	Discovery (CCGA)
NC83	Former smoker	NA	Not Available	Passed	Discovery (CCGA)

NC84	Non-smoker	NA	Not Available	Passed	Discovery (CCGA)
NC85	Non-smoker	NA	Not Available	Passed	Discovery (CCGA)
NC86	Non-smoker	NA	Not Available	Passed	Discovery (CCGA)
NC87	Former smoker	NA	Not Available	Passed	Discovery (CCGA)
NC88	Former smoker	NA	Not Available	Passed	Discovery (CCGA)
NC89	Former smoker	NA	Not Available	Passed	Discovery (CCGA)
NC90	Former smoker	NA	Not Available	Passed	Discovery (CCGA)
NC91	Non-smoker	NA	Not Available	Passed	Discovery (CCGA)
NC92	Former smoker	NA	Not Available	Passed	Discovery (CCGA)
NC93	Non-smoker	NA	Not Available	Passed	Discovery (CCGA)
NC94	Former smoker	NA	Not Available	Passed	Validation
NC95	Non-smoker	NA	Not Available	Passed	Validation
NC96	Former smoker	NA	Not Available	Passed	Validation
NC97	Non-smoker	NA	Not Available	Passed	Validation
NC98	Non-smoker	NA	Not Available	Passed	Validation
NC99	Non-smoker	NA	Not Available	Passed	Validation
NC100	Non-smoker	NA	Not Available	Passed	Validation
NC101	Non-smoker	NA	Not Available	Passed	Validation
NC102	Non-smoker	NA	Not Available	Passed	Validation
NC103	Non-smoker	NA	Not Available	Passed	Validation
NC104	Former smoker	NA	Not Available	Passed	Validation
NC105	Non-smoker	NA	Not Available	Passed	Validation

NC106	Non-smoker	NA	Not Available	Passed	Validation
NC107	Non-smoker	NA	Not Available	Passed	Validation
NC108	Non-smoker	NA	Not Available	Passed	Validation
NC109	Non-smoker	NA	Not Available	Passed	Validation
NC110	Non-smoker	NA	Not Available	Passed	Validation
NC111	Former smoker	NA	Not Available	Passed	Validation
NC112	Non-smoker	NA	Not Available	Passed	Validation
NC113	Non-smoker	NA	Not Available	Passed	Validation
NC114	Non-smoker	NA	Not Available	Passed	Validation
NC115	Non-smoker	NA	Not Available	Passed	Validation
NC116	Non-smoker	NA	Not Available	Passed	Validation
NC117	Missing	NA	Not Available	Passed	Validation
NC118	Former smoker	NA	Not Available	Passed	Validation
NC119	Former smoker	NA	Not Available	Passed	Validation
NC120	Non-smoker	NA	Not Available	Passed	Validation
NC121	Non-smoker	NA	Not Available	Passed	Validation
NC122	Former smoker	NA	Not Available	Passed	Validation
NC123	Current smoker	NA	Not Available	Passed	Validation
NC124	Non-smoker	NA	Not Available	Passed	Validation
NC125	Non-smoker	NA	Not Available	Passed	Validation

BrCa, breast cancer; CCGA, Circulating Cell-free Genome Atlas study (NCT02889978); HER2+/-, human epidermal growth factor receptor 2-positive/negative; HR+/–, hormone receptor positive/negative; LuCa, lung cancer; NA, not applicable; NC, non-cancer; TNBC, triple-negative breast cancer.

Supplementary Table 6. Annotation of cell-free RNA biomarker genes

	CCGA Discovery Cohort			CCGA Validation Cohort		
**Gene**	**Biomarker Type**	**Cancer**	**Subtype Specificity**	**Median RPM**	**Detected**	**Differentially Expressed (vs Non-Cancer)**
SLC34A2	DCB	Lung	Adenocarcinoma	0	Detected	Lung
GABRG1	DCB	Lung	NA	0	Detected	No
ROS1	DCB	Lung	Adenocarcinoma	0	Detected	Lung
AGR2	DCB	Lung	NA	0	Detected	Lung/Breast
GNAT3	DCB	Lung	NA	0	Detected	No
SFTPA2	DCB	Lung	Adenocarcinoma	0	Detected	Lung
MUC5B	DCB	Lung	NA	0	Detected	Lung
SFTA3	DCB	Lung	Adenocarcinoma	0	Detected	Lung
SMIM22	DCB	Lung	NA	0	Detected	Lung/Breast
CXCL17	DCB	Lung	Adenocarcinoma	0	Detected	Lung
BPIFA1	DCB	Lung	Adenocarcinoma	0	Detected	Lung
WFDC2	DCB	Lung/Breast	NA	0	Detected	Lung/Breast
CSN1S1	DCB	Breast	NA	0	Detected	No
FABP7	DCB, HeteroDE	Breast	TNBC	0	Not Detected	No

OPN1SW	DCB	Breast	NA	0	Not Detected	No
SCGB2A2	DCB, HeteroDE	Breast	HR+	0	Detected	Breast
LALBA	DCB	Breast	NA	0	Detected	No
CASP14	DCB, HeteroDE	Breast	NA	0	Detected	No
KLK5	DCB	Breast	NA	0	Detected	No
CRABP2	HeteroDE	Breast	NA	>0	Detected	Lung/Breast
VGLL1	HeteroDE	Breast	NA	0	Detected	Lung/Breast
SERPINB5	HeteroDE	Breast	NA	0	Detected	Lung
TFF1	HeteroDE	Breast	NA	0	Detected	Lung/Breast

CCGA, Circulating Cell-free Genome Atlas study (NCT02889978); cfRNA, cell-free RNA; DCB, dark channel biomarker; HeteroDE, heterogeneous differential expression; HR+, hormone receptor-positive; RPM, reads per million; TNBC, triple-negative breast cancer.

Supplementary Table 7. Positive control genes in targeted panel

AKT1	*MAP2K1*	*FGFR1*	*IDH1*	*PIK3R1*
BRAF	*NRAS*	*FGFR2*	*IDH2*	*MET*
CDKN2A	*PIK3CA*	*FGFR3*	*KDR*	*ALK*
DDR2	*PTEN*	*FGFR4*	*KIT*	*NTRK1*
EGFR	*STK11*	*GNA11*	*MLH1*	*RET*
ERBB2	*TP53*	*GNAQ*	*PDGFRA*	*FOXO1*
KRAS	*CSF1R*	*HRAS*	*PDGFRB*	
