# Supplementary material for: A comprehensive characterization of the cell-free transcriptome reveals tissue- and subtype-specific biomarkers for cancer detection
Source: Nat Commun. 2021 Apr 21;12:2357. doi: 10.1038/s41467-021-22444-1 (PMC8060291; doi:10.1038/s41467-021-22444-1)
Supplement: Supplementary file 3 — Reporting Summary [file 41467_2021_22444_MOESM3_ESM.pdf]

nature research

Corresponding author(s): Matthew H. Larson

Last updated by author(s): Feb 9, 2021

Reporting Summary

Nature Research wishes to improve the reproducibility of the work that we publish. This form provides structure for consistency and transparency in reporting. For further information on Nature Research policies, see our Editorial Policies and the Editorial Policy Checklist.

Statistics

For all statistical analyses, confirm that the following items are present in the figure legend, table legend, main text, or Methods section.

n/a	Confirmed	
☐	☒	The exact sample size (*n*) for each experimental group/condition, given as a discrete number and unit of measurement
☐	☒	A statement on whether measurements were taken from distinct samples or whether the same sample was measured repeatedly
☐	☒	The statistical test(s) used AND whether they are one- or two-sided<br>*Only common tests should be described solely by name; describe more complex techniques in the Methods section.*
☐	☒	A description of all covariates tested
☐	☒	A description of any assumptions or corrections, such as tests of normality and adjustment for multiple comparisons
☐	☒	A full description of the statistical parameters including central tendency (e.g. means) or other basic estimates (e.g. regression coefficient) AND variation (e.g. standard deviation) or associated estimates of uncertainty (e.g. confidence intervals)
☐	☒	For null hypothesis testing, the test statistic (e.g. *F*, *t*, *r*) with confidence intervals, effect sizes, degrees of freedom and *P* value noted<br>*Give P values as exact values whenever suitable.*
☒	☐	For Bayesian analysis, information on the choice of priors and Markov chain Monte Carlo settings
☒	☐	For hierarchical and complex designs, identification of the appropriate level for tests and full reporting of outcomes
☐	☒	Estimates of effect sizes (e.g. Cohen's *d*, Pearson's *r*), indicating how they were calculated

Our web collection on statistics for biologists contains articles on many of the points above.

Software and code

Policy information about availability of computer code

Data collection	Sequencing instrument control software for the HiSeq sequencing system was provided by Illumina.
Data analysis	All code and scripts are available for academic use at our Github repository: https://github.com/grailbio-publications/Larson_cfRNA_DarkChannelBiomarkers. Statistical analyses were performed using the R environment for statistical computing (v3.6.2). R packages used in the analyses (including version numbers) can be found in the github repository at https://github.com/grailbio-publications/Larson_cfRNA_DarkChannelBiomarkers/blob/master/packrat/packrat.lock.<br><br>A detailed description of the pipeline and assumptions made for transcript quantification is provided in the Methods and Supplementary Methods. All patient cfRNA-seq libraries were sequenced on HiSeq flowcells. Raw reads were aligned to GENCODE v19 primary assembly with all transcripts using STAR version 2.5.3a. Duplicate sequence reads were detected and removed using custom software based on genomic alignment position and non-random UMI sequences. Sets of reads sharing alignment position and UMIs were error corrected via multiple sequence alignment of member reads and a single consensus sequence/alignment was generated. All downstream analyses relied on the use of “strict RNA reads,” defined as read pairs where at least 1 read overlapped an exon-exon junction.<br><br>HeteroDE is a statistical method developed to identify biomarker genes from highly heterogeneous plasma cfRNA samples. It is packaged into the cellfreetranscriptome R package hosted on our Github repository. HeteroDE models the abundance of RNA transcripts in plasma using a negative binomial generalized linear model (NB-GLM). Tumor content was used as the covariate in the model to account for the influence from both the gene expression in the tumor tissue and the tumor shedding rate. The P value of the NB-GLM was computed using the glm.nb function in the MASS package (v7.3-51.4) in R. The P value cut-off was set to 0.05 for the biomarker candidates.

For manuscripts utilizing custom algorithms or software that are central to the research but not yet described in published literature, software must be made available to editors and reviewers. We strongly encourage code deposition in a community repository (e.g. GitHub). See the Nature Research guidelines for submitting code & software for further information.

Data

Policy information about availability of data

All manuscripts must include a data availability statement. This statement should provide the following information, where applicable:
- Accession codes, unique identifiers, or web links for publicly available datasets
- A list of figures that have associated raw data
- A description of any restrictions on data availability

All sequencing data have been deposited at the European Genome-phenome Archive (EGA) which is hosted at the European Bioinformatics Institute and the Centre for Genomic Regulation, and is publicly available under accession number EGAS00001004704 (https://www.ebi.ac.uk/ega/studies/EGAS00001004704). Data access can be obtained through a request to the GRAIL Data Access Committee (https://www.ebi.ac.uk/ega/dacs/EGAC00001001769).

Tissue-specific gene files for lung and breast tissues were downloaded from the Human Protein Atlas website (www.proteinatlas.org, version 18.1). Tissue deconvolution was performed using gene expression data downloaded from the GTEx Portal website (www.gtexportal.org/home/datasets, GTEx analysis V4). TCGA tumor tissue expression data are publicly available through the TCGA portal (https://portal.gdc.cancer.gov).

The remaining data are available within the article, Supplementary Information, or available from the authors upon request.

Field-specific reporting

Please select the one below that is the best fit for your research. If you are not sure, read the appropriate sections before making your selection.

☒ Life sciences ☐ Behavioural & social sciences ☐ Ecological, evolutionary & environmental sciences

For a reference copy of the document with all sections, see nature.com/documents/nr-reporting-summary-flat.pdf

Life sciences study design

All studies must disclose on these points even when the disclosure is negative.

Sample size	For the discovery cohort, we selected stage III breast and lung cancer samples from the CCGA study (NCT02889978). Stage III samples were selected to maximize signal in the blood while avoiding confounding signal from potential secondary metastases. We required that the selected patients had at least 2 tubes of unprocessed grade 1-2 plasma (no hemolysis), with 6-8 mL of plasma per patient. We further required that selected patients had matched cfDNA sequencing data from previous GRAIL studies. All cancer patients (47 breast, 32 lung) that matched these criteria were utilized for this study in order to maximize the potential for biomarker discovery. We also selected participants with no known history of cancer to comprehensively characterize cfRNA in non-cancer patients. 93 samples were selected to appropriately match the cancer samples for age, sex, and ethnicity. This study was not powered to assess the clinical performance of cfRNA for cancer classification.<br><br>For the validation cohort, we obtained 38 breast cancer and 18 lung cancer samples from Discovery Life Sciences. An attempt was made to validate cfRNA biomarkers in a separate cancer cohort of approximately equal size to the discovery cancer cohort, and was limited based on sample availability. 32 age-matched non-cancer samples were included as controls of expression in patients without cancer.
Data exclusions	One assay metric and 3 pipeline metrics were pre-established as “red flags” and were used to exclude samples with poor metrics. The assay metric measured whether samples had sufficient material for sequencing, and the pipeline metrics were sequencing depth, RNA purity, and cross-sample contamination. 1 sample was flagged due to insufficient material for sequencing, and 6 samples were removed from further analyses due to low RNA purity.
Replication	Each patient sample was used in its entirety during RNA-seq library preparation. As such, no replicates were performed for a given patient. Our CCGA discovery cohort included cancer (stage III breast [n=46] and lung [n=30]) and non-cancer (n=89) participants. The cfRNA biomarkers identified in the CCGA discovery cohort were validated using a separate cohort of breast (n=38) and lung (n=18) cancer plasma samples, and 32 age-matched non-cancer samples obtained from a commercial vendor (Discovery Life Sciences). All patient samples were used in their entirety without replication. Only 1 patient sample in this study failed to yield enough library for sequencing.
Randomization	Once the cancer and non-cancer samples were selected based on the above criteria, a randomization function in R was used to ensure that the entire group is fully randomized. The group was then divided into batches that have random mixtures of cancer types (cancer and non-cancer samples) for sample processing.
Blinding	Patient samples were assigned anonymized IDs that obscured patient status during sample processing. QC analysis and data exclusions were applied before unblinding of patient samples. Blinding was not relevant for data analysis, as the study was designed to characterize the cell-free transcriptome in cancer and non-cancer patients.

Reporting for specific materials, systems and methods

We require information from authors about some types of materials, experimental systems and methods used in many studies. Here, indicate whether each material, system or method listed is relevant to your study. If you are not sure if a list item applies to your research, read the appropriate section before selecting a response.

Materials & experimental systems

n/a	Involved in the study
☒	☐ Antibodies
☒	☐ Eukaryotic cell lines
☒	☐ Palaeontology and archaeology
☒	☐ Animals and other organisms
☐	☒ Human research participants
☐	☒ Clinical data
☒	☐ Dual use research of concern

Methods

n/a	Involved in the study
☒	☐ ChIP-seq
☒	☐ Flow cytometry
☒	☐ MRI-based neuroimaging

Human research participants

Policy information about studies involving human research participants

Population characteristics

The demographic characteristics of the discovery and validation cohorts are presented in Supplementary Tables 1-4.

Among the 47 patients in the CCGA breast cancer discovery cohort, 14 (30%) were hormone receptor-positive and HER2-negative, 11 (23%) were hormone receptor-positive and HER2-positive, 5 (11%) were hormone receptor-negative and HER2-positive, 14 (30%) were triple negative breast cancers, and 3 (6%) were missing subtype information. All patients were diagnosed with stage III breast cancer at the time of sample collection. The median age was 53 (IQR range 41-60), and and 47 (100%) were female.

Among the 32 patients in the CCGA lung cancer cohort, 11 (34%) were adenocarcinomas, 10 (31%) were squamous cell carninomas, 9 (28%) were small cell lung cancers, 1 (3%) was a lung carcinoid, and 1 (3%) was missing subtype information. , All patients were diagnosed with stage III lung cancer at the time of sample collection. The median age was 66 (IQR range 60-73), and 18 (56%) were female.

The CCGA non-cancer cohort included 93 non-cancer controls with no known current or prior diagnosis of cancer. The median age was 62 (IQR range 55-69), and 68 (73%) were female.

The breast cancer validation cohort was comprised of 38 patients (stages I [n=5, 13%], II [n=8, 21%], III [n=10, 26%], IV [n=15, 40%]). The median age was 58 (IQR range 40.5-75.5) and 38 (100%) were female.

The lung cancer validation cohort was comprised of 18 patients (stages I [n=1, 5.5%], II [n=1, 5.5%], III [n=7, 39%], IV [n=9, 50%]. The median age was 63.5 (IQR range 54-73) and 12 (67%) were female.

The non-cancer validation cohort included 32 non-cancer controls with no known current or prior diagnosis of cancer. The median age was 62 (IQR range 55-69), and 28 (87.5%) were female.

Recruitment

Inclusion Criteria for Non-Cancer Arm Participants:
- Age 20 years or older
- Able to provide a written informed consent

Exclusion Criteria for Non-Cancer Arm Participants:
- Known current or prior diagnosis of cancer except non-melanoma skin cancer
- Oral or IV corticosteroid use in past 14 days prior to blood draw
- Pregnancy (by self-report)
- Current febrile illness
- Acute exacerbation or flare of an inflammatory condition requiring escalation in medical therapy within 14 days prior to blood draw
- Recipient of organ transplant or prior non-autologous (allogeneic) bone marrow or stem cell transplant
- Poor health status or unfit to tolerate blood draw

Inclusion Criteria for Cancer Arm Participants:
- Age 20 years or older
- Able to provide a written informed consent
- Have either of the following:

A. Confirmed cancer diagnosis. Any stage I-IV, as well as carcinoma in situ (CIS) within 90 days prior to or up to 42 days after study blood draw, based upon assessment of a pathological specimen

OR

B. A high suspicion for a cancer diagnosis by clinical and/or radiological assessment, with planned biopsy or surgical resection to establish a definitive diagnosis within 6 weeks (42 days) after study blood draw

	Exclusion Criteria for Cancer Arm Participants:<br>- Known prior diagnosis of cancer except non-melanoma skin cancer<br>- Currently receiving, or ever received, any of the following therapies to treat their current cancer: surgical management of the cancer beyond that required to establish the cancer diagnosis; local, regional or systemic chemotherapy including chemoembolization; targeted therapy, immunotherapy including cancer vaccines; hormone therapy; or radiation therapy<br>- Pregnancy (by self-report)<br>- Current febrile illness<br>- Acute exacerbation or flare of an inflammatory condition requiring escalation in medical therapy within 14 days prior to blood draw<br>- Recipient of organ transplant or prior non-autologous (allogeneic) bone marrow or stem cell transplant<br>- Poor health status or unfit to tolerate blood draw
Ethics oversight	The protocol was reviewed and approved by the Institutional Review Board (IRB) or Independent Ethics Committee (IEC) for each of the 142 participating sites (full list can be found at http://clinicaltrials.gov/ct2/show/NCT02889978). IRB Approval Letters, IRB Rosters, and Informed Consent Forms for each site are available in the Trial Master File and are available upon request.<br><br>Informed written consent was obtained for each participant prior to sample collection. The Informed Consent Form contains the following statement: "The results of the study may be published in scientific journals and presented at medical meetings. The study doctor, study staff, and sponsor may make data, results, or biological samples from the study available in publicly accessible databases or provide them to other researchers for use in other research projects. If the data, results, or biological samples from this study are made public or provided to other researchers, information that directly identifies you will not be used.”

Note that full information on the approval of the study protocol must also be provided in the manuscript.

Clinical data

Policy information about clinical studies

All manuscripts should comply with the ICMJE guidelines for publication of clinical research and a completed CONSORT checklist must be included with all submissions.

Clinical trial registration	NCT02889978
Study protocol	http://clinicaltrials.gov/ct2/show/NCT02889978
Data collection	Clinical information, demographics, and medical data relevant to cancer status are collected from all participants and their medical record at baseline (time of biospecimen collection).
Outcomes	The primary outcome of this study was to collect and study clinically-annotated biospecimens, specifically peripheral blood and contemporary tumor tissue when available, to characterize cell-free nucleic acid profiles from deep sequencing and to estimate the population heterogeneity in two arms of the study (cancer vs non-cancer).
